# Induction and inhibition of the pan-nuclear gamma-H2AX response in resting human peripheral blood lymphocytes after X-ray irradiation

**DOI:** 10.1038/cddiscovery.2016.11

**Published:** 2016-03-21

**Authors:** D Ding, Y Zhang, J Wang, X Zhang, Y Gao, L Yin, Q Li, J Li, H Chen

**Affiliations:** 1 Department of Radiation Biology, Institute of Radiation Medicine, Fudan University, Shanghai, China; 2 Clinical Department at Renji Hospital, Shanghai Jiao Tong University School of Medicine, Shanghai, China

## Abstract

Human peripheral blood lymphocytes (HPBLs) are one of the most sensitive cells to ionizing radiation (IR) in the human body, and IR-induced DNA damage and functional impairment of HPBLs are the adverse consequences of IR accidents and major side effects of radiotherapy. Phosphorylated H2AX (*γ*H2AX) is a sensitive marker for DNA double-strand breaks, but the role and regulation of the pan-nuclear *γ*H2AX response in HPBLs after IR remain unclear. We herein demonstrated that the pan-nuclear *γ*H2AX signals were increased in a time- and dose-dependent manner, colocalized with >94% of TUNEL apoptotic staining, and displayed a typical apoptotic pattern in resting HPBLs after low LET X-ray IR. In addition, the X-irradiation-induced pan-nuclear p-ATM and p-DNA-PKcs responses also occurred in resting HPBLs, and were colocalized with 92–95% of TUNEL staining and 97–98% of the pan-nuclear *γ*H2AX signals, respectively, with a maximum at 6 h post irradiation, but disappeared at 24 h post irradiation. Moreover, ATM/DNA-PKcs inhibitor KU55933, p53 inhibitor PFT-*μ* and pan-caspase inhibitor ZVAD-fmk significantly decreased X-irradiation-induced pan-nuclear *γ*H2AX signals and TUNEL staining, protected HPBLs from apoptosis, but decreased the proliferative response to mitogen in X-irradiated HPBLs. Notably, whereas both KU55933 and PFT-*μ* increased the IR-induced chromosome breaks and mis-repair events through inhibiting the formation of p-ATM, p-DNA-PKcs and *γ*H2AX foci in X-irradiated HPBLs, the ZVAD-fmk did not increase the IR-induced chromosomal instability. Taken together, our data indicate that pan-nuclear *γ*H2AX response represents an apoptotic signal that is triggered by the transient pan-nuclear ATM and DNA-PKcs activation, and mediated by p53 and pan-caspases in X-irradiated HPBLs, and that caspase inhibitors are better than ATM/DNA-PKcs inhibitors and p53 inhibitors to block pan-nuclear *γ*H2AX response/apoptosis and protect HPBLs from IR.

## Introduction

In coping with ionizing radiation (IR)-induced double strand breaks (DSBs), mammalian cells promptly initiate the DNA damage response (DDR) to ensure efficient and accurate repair of damaged DNA and cell survival.^1^ It is commonly believed that a key step in the response to DSBs is histone variant H2AX phosphorylation at serine 139 (S139) in the megabase chromatin region flanking the break to provide a docking site for DNA repair factors, which form visible *γ*H2AX immunofluorescent foci in mammals.^2,3^ Ataxia telangiectasia mutated (ATM) and DNA-dependent protein kinase catalytic subunit (DNA-PKcs) are two primary kinases that phosphorylate H2AX at S139 in response to IR-induced DSBs.^4–6^ Moreover, ATM also phosphorylates and activates the tumor suppressor p53 to induce cell cycle arrest, DNA repair or apoptosis by transactivating downstream target genes,^7,8^ and DNA-PKcs is dispensable for p53 activation.^9–13^ In addition to its role in transcription regulation, p53 also functions in a transcriptionally independent manner in DNA repair^14^ and apoptosis.^15,16^ The activation of the caspase family of cysteine proteases has been shown as an extremely important step in the execution of p53-mediated transcription-dependent and -independent apoptosis.^17^

Apart from IR-induced formation of *γ*H2AX foci, the induction of pan-nuclear *γ*H2AX signals has been demonstrated as a cellular reaction to IR and as a consequence of various regulation mechanisms.^18–20^ Cook *et al.*^18^ have reported that H2AX phosphorylation at tyrosine 142 (Y142) induces an apoptotic response instead of a damage repair response to genotoxic stress by preventing the binding of the damage repair factors to phospho-serine 139 of *γ*H2AX and promoting the effective recruitment of pro-apoptotic factors to H2AX. Moreover, H2AX phosphorylation at Y142 produces visible whole-nuclear *γ*H2AX immunofluorescence staining instead of foci in TUNEL-positive mouse embryonic fibroblasts exposed to 100 Gy of irradiation.^18^ However, the relationship between pan-nuclear *γ*H2AX response and radiation quality remains unclear. Markova *et al.*^19^ observed the *γ*-irradiation-induced pan-nuclear *γ*H2AX response in HPBLs, but it was unknown whether the pan-nuclear *γ*H2AX response was related to apoptosis. In addition, it was demonstrated that localized heavy ion irradiation-induced clustered DNA damage in sub-nuclear regions induced the transient nuclear-wide H2AX phosphorylation in undamaged chromatin in mammalian cells, including human fibroblasts, the U2-OS tumor cells, mouse embryonic fibroblast cells and hamster ovary cells; however, none of the neighboring un-irradiated cells showed a nuclear-wide *γ*H2AX response, and targeted cytoplasmic irradiation with particles also did not induce the nuclear-wide *γ*H2AX response.^20^ It has been proposed that the particle-induced pan-nuclear *γ*H2AX response is caused by ATM and DNA-PK activity distant to sites of DNA damage and is not related to either apoptosis or DSB repair.^20^ The nuclear-wide kinase activity of ATM and DNA-PK may be elicited by dense IR-induced small DNA fragments^21^ or by diffusion of active kinases from DNA DSBs. In addition, pan-nuclear *γ*H2AX signals were induced by other stressors via various regulation mechanisms. It has been reported that tumor necrosis factor-related apoptosis-inducing ligand (TRAIL), staurosporine, etoposide and ultraviolet irradiation induce the pan-nuclear *γ*H2AX response associated with apoptosis in human cancer cells,^22^ mouse fibroblasts, Chinese hamster ovary cells, human fibroblasts^23–25^ and resting human T cells.^26^ The pan-nuclear *γ*H2AX response can be caused by replication-mediated DSBs in cancer cells treated with camptothecin,^27^ indenoisoquinolines^28^ or gemcitabine,^29^ which are not related to apoptotic DNA fragmentation. Moreover, small DNA molecules, which mimic DNA DSBs (called D bait) and disorganize the DNA repair system in cancer cells,^30,31^ and adeno-associated virus replication in infected host cells, which could be explained as a reaction to the single-stranded DNA part of the virus that compromises DNA repair, also can induce the pan-nuclear *γ*H2AX response.^32,33^ The changes in chromatin structure after hypotonic treatment are sufficient to elicit extensive pan-nuclear *γ*H2AX formation in the absence of DNA strand breaks, which are not related to apoptosis.^34^

Human peripheral blood lymphocytes (HPBLs) are extremely sensitive to IR and represent the most common target cells for biological dosimetric,^35^ radiosensitive^36^ and immune function studies.^37,38^ *γ*H2AX foci have been shown to be the most sensitive dosimeter and radiosensitive indicator.^39^ HPBLs are non-dividing cells in the G0 phase of the cell cycle, and can proliferate to generate the immune response if stimulated. Therefore, HPBLs are able to efficiently repair DSBs to prevent apoptosis and mutations. Low LET *γ*-ray irradiation induces the activation of DDR proteins through phosphorylation of ATM at Ser 1981, DNA-PKcs at tyrosine 2609 and H2AX at Ser 139 to form DNA repair foci in HPBLs.^19,40,41^ If the DNA damage is not repaired after IR, HPBLs undergo p53-dependent apoptosis through the caspase cascade,^42–47^ and subsequently, functional inhibition of HPBLs may result in immune suppression in individuals exposed to acute or chronic IR at high doses and in radio/chemotherapy patients.^48,49^ Therefore, it is of interest to understand the relationships among pan-nuclear *γ*H2AX signals, apoptosis, DSB repair, chromosomal aberration and proliferative function in resting HPBLs exposed to low LET X-irradiation, and to explore the pharmacological approaches to protect HPBLs against IR-induced pan-nuclear *γ*H2AX response/apoptosis. In this study, we demonstrated that X-ray-induced pan-nuclear *γ*H2AX response represents an apoptotic signal in resting HPBLs, and is mediated by transient pan-nuclear ATM and DNA-PKcs activation and dependent on p53 and caspase activation. Our studies suggest that caspase inhibitors are better than ATM/DNA-PKcs inhibitors and p53 inhibitors to block pan-nuclear *γ*H2AX response/apoptosis and protect HPBLs from IR.

## Results

### The kinetic patterns of the pan-nuclear responses are different from that of the DDR foci in resting HPBLs after X-irradiation

**Figure 1:**
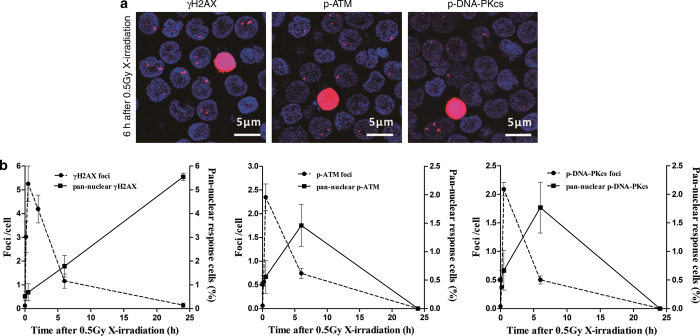
Distinct kinetic patterns between the pan-nuclear responses and the repair foci of *γ*H2AX, p-ATM and p-DNA-PKcs. (**a**) Representative images of X-irradiation-induced pan-nuclear signals and foci of *γ*H2AX, p-ATM and p-DNA-PKcs in resting HPBLs at 6 h after 0.5 Gy X-ray exposure. ×60 magnification, ×1.0 zoom. (**b**) HPBLs were irradiated with 0.5 Gy X-ray, and the pan-nuclear signals and foci of *γ*H2AX (left panel), p-ATM (middle panel) and p-DNA-PKcs (right panel) were examined at the indicated time points after irradiation and quantified by counting 1000–5000 HPBLs from each sample. *γ*H2AX, p-ATM and p-DNA-PKcs were labeled in red, and the nuclei were stained in blue with DAPI. Data are presented as the means±S.D. of four to six donors.

As shown in Figure 1a, we detected the nuclear-wide *γ*H2AX, p-ATM and p-DNA-PKcs responses and the *γ*H2AX, p-ATM and p-DNA-PKcs foci in resting HPBLs after X-irradiation. Importantly, the kinetic patterns of the pan-nuclear responses are different from that of the DDR foci (Figure 1b). For the *γ*H2AX signals, although the percentages of pan-nuclear *γ*H2AX-positive cells increased steadily after X-irradiation, the frequencies of the *γ*H2AX foci peaked at 30 min after X-irradiation, decreased rapidly during 6 h post irradiation and was reduced to approximately 8% of the peak value at 24 h post irradiation (Figure 1b, left panel). For the p-ATM and p-DNA-PKcs signals, the kinetic patterns of the p-ATM and p-DNA-PKcs foci were similar to that of the *γ*H2AX foci, but the percentages of pan-nuclear p-ATM- and p-DNA-PKcs-positive cells peaked at 6 h post irradiation and decreased to zero at 24 h post irradiation (Figure 1b, middle and right panel). These data suggest that the nuclear-wide *γ*H2AX, p-ATM and p-DNA-PKcs responses are not related to DSB repair in resting HPBLs after X-irradiation.

### X-irradiation-induced pan-nuclear *γ*H2AX, p-ATM and p-DNA-PKcs signals are colocalized with TUNEL apoptotic staining in resting HPBLs

**Figure 2:**
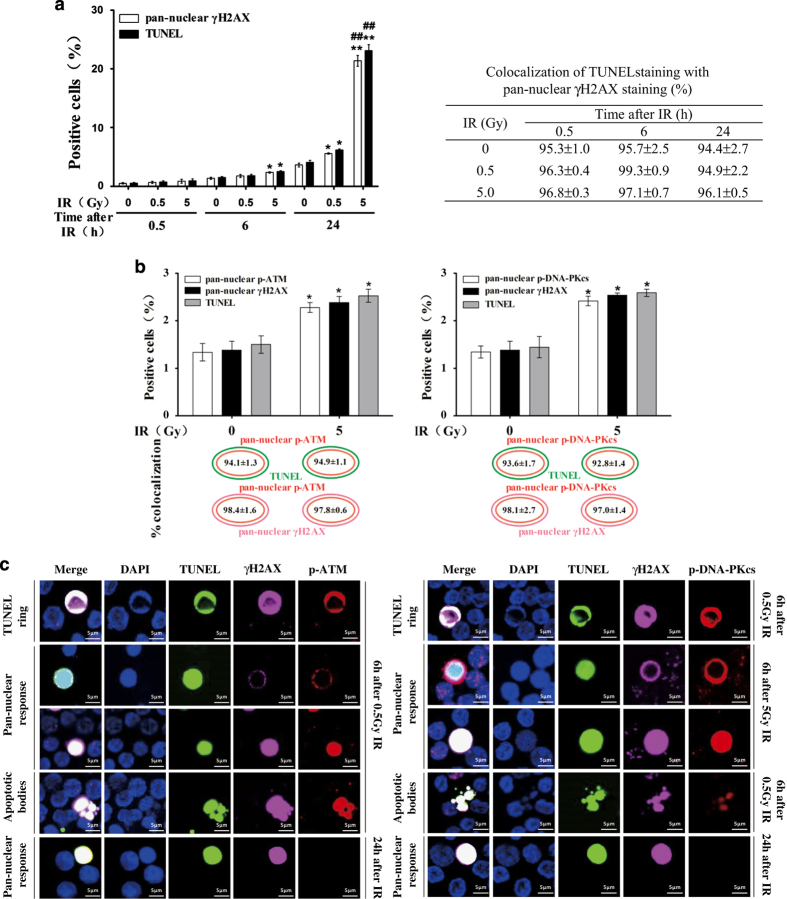
Relationship between pan-nuclear *γ*H2AX, p-ATM and p-DNA-PKcs responses and apoptosis in resting HPBLs exposed to X-ray irradiation. (**a**) HPBLs were irradiated with 0.5 and 5 Gy X-ray. The pan-nuclear *γ*H2AX, TUNEL-positive cells (left panel) and their colocalization (right panel) were examined at the indicated time points after irradiation. (**b**) HPBLs were irradiated with 5 Gy X-ray. The pan-nuclear p-ATM with the pan-nuclear *γ*H2AX and TUNEL-positive cells, pan-nuclear p-DNA-PKcs with the pan-nuclear *γ*H2AX and TUNEL-positive cells and their colocalization were examined at 6h after irradiation. (**c**) Representative images of X-irradiation-induced pan-nuclear *γ*H2AX, p-ATM, p-DNA-PKcs and TUNEL staining and their colocalization in resting HPBLs at the indicated time points after irradiation. *γ*H2AX was labeled in infrared, p-ATM and p-DNA-PKcs in red and TUNEL in green, and nuclei were stained blue with DAPI. A total of 5000 HPBLs from each sample were examined to calculate the percentage of pan-nuclear *γ*H2AX, p-ATM, p-DNA-PKcs and TUNEL-positive cells. ×60 magnification, ×1.0 zoom. Data shown in panel (**a**) and panel (**b**) are presented as the means±S.D. of six donors. *P<0.05 and ^**^ *P*<0.01 compared with corresponding non-irradiated HPBLs at 6 or 24 h post irradiation; ^##^ *P*<0.01 compared with corresponding HPBLs exposed to 0.5 Gy X-ray at 24 h post irradiation.

It was reported that pan-nuclear formation of *γ*H2AX in undamaged chromatin induced by high-LET localized ion irradiation was not related to apoptosis in human fibroblasts, tumor cells and hamster ovary cells.^20^ However, we found that the kinetics of the increase of the pan-nuclear *γ*H2AX response was similar to that of apoptotic induction at 0.5, 6 and 24 h post irradiation (0.5 or 5.0 Gy) in HPBLs. Moreover, the percentage of TUNEL-positive cells was similar to the percentage of pan-nuclear *γ*H2AX-positive cells at each time point after the same dose radiation (Figure 2a, left panel). More importantly, 94–99% of the TUNEL-positive cells also displayed pan-nuclear *γ*H2AX staining in a dose- and time-independent manner (Figure 2a, right panel; Figure 2c), indicating that pan-nuclear *γ*H2AX staining was associated with X-irradiation-induced apoptosis in resting HPBLs.

Furthermore, pan-nuclear p-ATM and p-DNA-PKcs signals were also colocalized with pan-nuclear *γ*H2AX or TUNEL staining at 6 h post irradiation in resting HPBLs. It was found that 93–95% of the TUNEL-positive cells also exhibited pan-nuclear p-ATM or p-DNA-PKcs staining at 6 h post irradiation (5 Gy) (Figures 2b and c). We further found that 97–98% of cells with pan-nuclear *γ*H2AX staining showed pan-nuclear p-ATM and p-DNA-PKcs staining (Figures 2b and c). Notably, HPBLs with pan-nuclear *γ*H2AX staining did not exhibit pan-nuclear p-ATM and p-DNA-PKcs staining at 24 h post irradiation (Figure 2c), suggesting that ATM and DNA-PKcs were only activated at early stages after irradiation.

According to the morphological signs of apoptosis progression such as apoptotic rings, pan-nuclear staining and apoptotic bodies, we observed three types of morphological characterization of the apoptotic *γ*H2AX response: *γ*H2AX ring versus TUNEL ring and very weak TUNEL pan-staining of the nucleus; pan-nuclear *γ*H2AX versus strong TUNEL pan-staining of the nucleus; and the *γ*H2AX pan-staining within apoptotic bodies. Notably, no DDR foci were observed in the pan-nuclear responses.

### X-irradiation-induced pan-nuclear *γ*H2AX responses and apoptosis in resting HPBLs are mediated by ATM and DNA-PKcs

**Figure 3:**
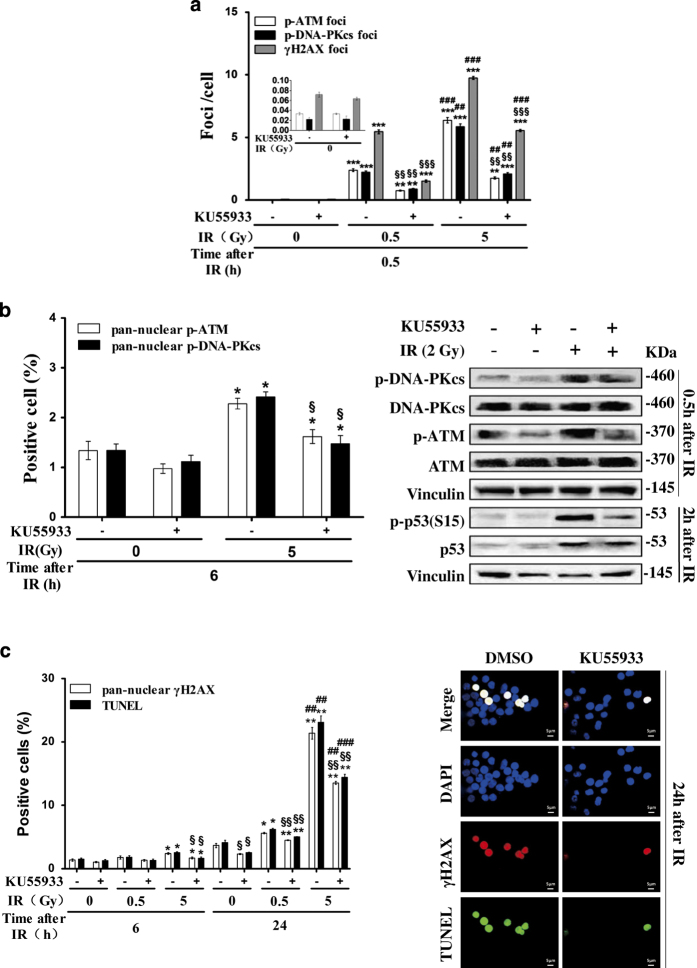
Inhibition of X-irradiation-induced pan-nuclear *γ*H2AX response and apoptosis in resting HPBLs by KU55933. (**a**) HPBLs were treated with 10 *μ*M KU55933 for 2 h before X-irradiation (0.5 or 5 Gy). *γ*H2AX, p-ATM and p-DNA-PKcs foci were measured in 500–1000 HPBLs from each sample. (**b**) HPBLs were treated with 10 *μ*M KU55933 for 2 h before X-irradiation (5 Gy). Pan-nuclear p-ATM and p-DNA-PKcs cells (left panel) were counted, and western blotting assays (right panel) were performed to examine the expression of p-ATM/ATM, p-DNA-PKcs/DNA-PKcs and p-p53/p53 proteins at the indicated time points after irradiation. (**c**) HPBLs were treated with 10 *μ*M KU55933 for 2 h before X-irradiation (0.5 or 5 Gy). Pan-nuclear *γ*H2AX and TUNEL-positive cells were measured, and their representative images were captured at 24 h after X-irradiation; *γ*H2AX was labeled in red and TUNEL in green, and nuclei were stained in blue with DAPI. A total of 2000–5000 HPBLs from each sample were examined to determine the percentage of the pan-nuclear *γ*H2AX, p-ATM, p-DNA-PKcs and TUNEL-positive cells. ×100 magnification, ×1.0 zoom. The values shown in panel (**a**), panel (**b**) and panel (**c**) represent the means±S.D. obtained from three to four donors. *P<0.05, **P<0.01 and ***P<0.001 compared with corresponding non-irradiated HPBLs; ^##^ *P*<0.01 and ^###^ *P*<0.001 compared with corresponding HPBLs exposed to 0.5 Gy X-ray irradiation with/without inhibitor treatment; ^§^ *P*<0.05, ^§§^ *P*<0.01 and ^§§§^ *P*<0.01 compared with corresponding HPBLs without inhibitor treatment.

We next investigated the role of ATM and DNA-PKcs in nuclear-wide H2AX phosphorylation and apoptosis. It was found that pretreatment with KU55933, a potent inhibitor of ATM/DNA-PKcs, resulted in significant inhibition of X-irradiation-induced *γ*H2AX, p-ATM and p-DNA-PKcs focus formation at 30 min post irradiation (0.5 or 5 Gy) in resting HPBLs (Figure 3a), suggesting that KU55933 inhibited X-irradiation-induced DSB repair. In addition, KU55933 significantly inhibited the pan-nuclear p-ATM and p-DNA-PKcs signals at 6 h post irradiation (5 Gy) (Figure 3b, left panel) in HPBLs. The inhibitory effects of KU55933 on p-ATM and p-DNA-PKcs expression were also demonstrated by western blotting analysis (Figure 3b, right panel). We found that KU55933 also inhibited X-irradiation-induced p53 phosphorylation (Figure 3b, right panel). Correspondingly, KU55933 inhibited the pan-nuclear *γ*H2AX signals and TUNEL apoptosis at 6 and 24 h post irradiation (0.5 or 5 Gy) in resting HPBLs (Figure 3c). Together, our results suggest that KU55933 decreases X-irradiation-induced pan-nuclear *γ*H2AX responses and apoptosis by inhibiting transient pan-nuclear p-ATM and p-DNA-PKcs responses and p53 phosphorylation in resting HPBLs.

### X-irradiation-induced pan-nuclear *γ*H2AX response and apoptosis in resting HPBLs were also mediated by p53 and pan-caspases

**Figure 4:**
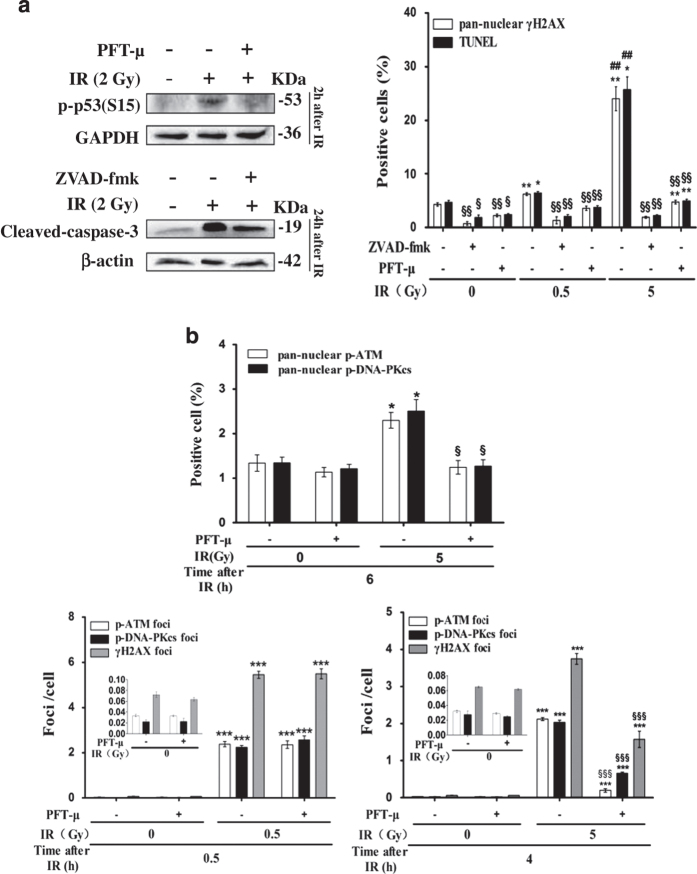
Inhibition of X-irradiation-induced pan-nuclear *γ*H2AX response and apoptosis in resting HPBLs by PFT-*μ* and ZVAD-fmk. (**a**) HPBLs were treated with 20 *μ*M PFT-*μ* for 5 h or 100 *μ*M ZVAD-fmk for 1 h before X-irradiation (2 Gy). The levels of p-p53 expression (left upper panel) and cleaved caspase-3 expression (left bottom panel) in HPBLs at indicated time points post irradiation were examined by western blotting. Furthermore, pan-nuclear *γ*H2AX and TUNEL-positive cells were counted at 24 h post irradiation. (**b**) HPBLs was treated with 20 *μ*M PFT-*μ* for 5 h before X-irradiation. Pan-nuclear p-ATM- and p-DNA-PKcs-positive cells were counted at 6 h post irradiation (upper panel). *γ*H2AX, p-ATM and p-DNA-PKcs foci were measured in HPBLs at 0.5 h (left bottom panel) and 4 h (right bottom panel) post irradiation. A total of 1000 HPBLs from each sample were examined for quantitation of the p-ATM, p-DNA-PKcs and *γ*H2AX foci, and 2000–5000 HPBLs from each sample were examined to calculate the percentage of pan-nuclear *γ*H2AX, p-ATM, p-DNA-PKcs and TUNEL-positive cells. The values shown in panel (**a**) and panel (**b**) represent the means±S.D. obtained from three to four donors. *P<0.05, **P<0.01 and ***P<0.001 compared with corresponding non-irradiated HPBLs with/without inhibitor treatment; ^##^ *P*<0.01 compared with corresponding HPBLs exposed to 0.5 Gy X-ray irradiation with/without inhibitor treatment; ^§^ *P*<0.05, ^§§^ *P*<0.01 and ^§§§^ *P*<0.01 compared with corresponding HPBLs without inhibitor treatment.

It has been shown that apoptosis of HPBLs is p53- and caspase-dependent.^26,43–47^ We next investigated the roles of p53 and caspases in nuclear-wide H2AX phosphorylation and apoptosis. It was found that pretreatment with the p53 inhibitor PFT-*μ* and pan-caspase inhibitor ZVAD-fmk significantly reduced X-irradiation-induced p53 phosphorylation and cleaved-caspase-3 expression in resting HPBLs (Figure 4a, left panel). Similar to the inhibitory effects of KU55933 on X-irradiation-induced pan-nuclear *γ*H2AX signals and TUNEL staining, pretreatment with PFT-*μ* or ZVAD-fmk also resulted in the significant suppression of X-irradiation-induced pan-nuclear *γ*H2AX signals and apoptosis at 24 h post irradiation (0.5 or 5 Gy) in resting HPBLs (Figure 4a, right panel), suggesting that p53 and caspases are involved in the regulation of the nuclear-wide *γ*H2AX response. Moreover, PFT-*μ* inhibited X-irradiation-induced pan-nuclear p-ATM and p-DNA-PKcs responses at 6 h post irradiation (5 Gy) and X-irradiation-induced p-ATM and p-DNA-PKcs focus formation at 4 h post irradiation, although it did not decrease X-irradiation-induced p-ATM and p-DNA-PKcs focus formation at 30 min post irradiation (Figure 4b), suggesting that p53 is involved in mediating the pan-nuclear p-ATM and p-DNA-PKcs responses, and has an important role in enhancing DSB repair in HPBLs after X-irradiation.

### Effects of KU55933, PFT-*μ* and ZVAD-fmk on X-irradiation-induced chromosomal aberrations and proliferative response in resting HPBLs

Although KU55933, PFT-*μ* and ZVAD-fmk protect HPBLs from X-irradiation-induced apoptosis, it is not clear whether they affect X-irradiation-induced genomic instability and protect HPBLs function. Table 1 shows that X-irradiation significantly induced chromosomal aberrations including breaks, dicentrics plus rings in resting HPBLs and increased the percentage of aberrant cells. KU55933 and PFT-*μ*, but not ZVAD-fmk, further significantly enhanced X-irradiation-induced chromosomal aberrations.

**Figure 5:**
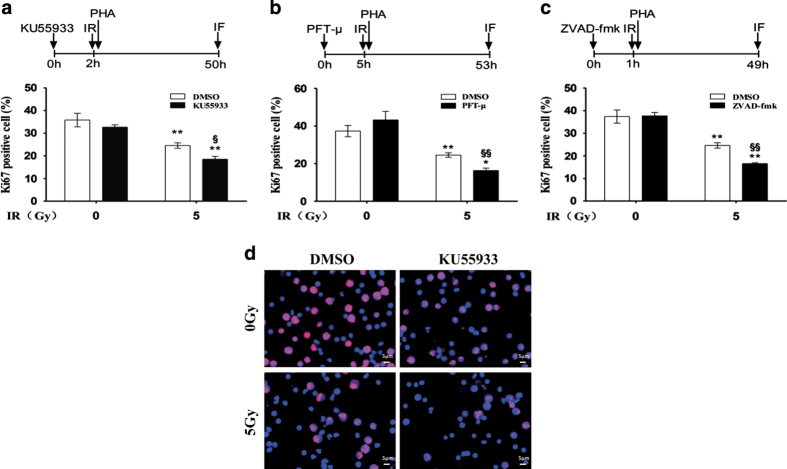
KU55933, PFT-*μ* and ZVAD-fmk inhibited the proliferative response to mitogen in resting HPBLs exposed to X-ray irradiation. HPBLs were treated with 10 *μ*M KU55933 for 2 h (**a**), 20 *μ*M PFT-*μ* for 5 h (**b**) or 100 *μ*M ZVAD-fmk for 1 h (**c**) followed by irradiation with 5 Gy X-ray, and were cultured with PHA and colcemid for 48 h. HPBLs were then collected and transformed HPBLs were detected using the Ki67 antibody. A total of 1000 HPBLs from each sample were examined to determine the proliferation ratio. Data are presented as the means±S.D. obtained from three donors. *P<0.05 and **P<0.01 compared with corresponding non-irradiated HPBLs with/without inhibitor treatment; ^§^ *P*<0.05 and ^§§^ *P*<0.01 compared with corresponding HPBLs without inhibitor treatment. (**d**) Representative images of HPBLs labeled with Ki67. The cells were treated with KU55933 and irradiated with X-ray as described above. Ki67 was labeled in red and nuclei were stained in blue with DAPI. ×40 magnification, ×1.0 zoom.

We investigated the impact of KU55933, PFT-*μ* and ZVAD-fmk on the proliferation of X-irradiated HPBLs. We found that the inhibitor (KU55933, PFT-*μ* or ZVAD-fmk) combined with X-irradiation resulted in a more significant decrease of the percentage of Ki67-positive cells than the inhibitor or X-irradiation alone in resting HPBLs, suggesting that KU55933, PFT-*μ* and ZVAD-fmk aggravated the inhibitory effect of X-irradiation on the proliferative response in resting HPBLs (Figures 5a–d).

## Discussion

Unlike the kinetics of localized ion-induced transient pan-nuclear *γ*H2AX response with a peak at ~1 h post irradiation, we demonstrated herein that X-irradiation-induced pan-nuclear *γ*H2AX response and apoptosis were increased in a dose- and time-dependent manner in resting HPBLs exposed to X-irradiation. This phenomenon is similar to the kinetics of the anticancer agent etoposide-induced pan-nuclear *γ*H2AX response and apoptosis in resting human T cells.^26^ More importantly, we provide direct evidence that the X-irradiation-induced pan-nuclear *γ*H2AX response is presented in the apoptotic HPBLs. The apoptotic *γ*H2AX pan-nuclear response is a novel feature of the low LET IR-induced apoptosis in HPBLs, and could serve as a biomarker to distinguish apoptotic cells from the DNA-damaged cells with a focal pattern and to monitor the extent of radiation injury and side effects of radiotherapy.

In contrast, X-irradiation-induced pan-nuclear p-ATM and p-DNA-PKcs responses in resting HPBLs were transient with a maximum response at 6 h post irradiation and complete disappearance at 24 h post irradiation. The transient X-irradiation-induced pan-nuclear p-ATM and p-DNA-PKcs responses are also presented in the apoptotic HPBLs. Moreover, ATM/DNA-PKcs inhibitor KU55933 not only inhibited X-irradiation-induced p-ATM and p-DNA-PKcs foci and the pan-nuclear p-ATM and p-DNA-PKcs responses but also suppressed the pan-nuclear *γ*H2AX response and apoptosis in resting HPBLs. DNA-PK can be strongly activated during apoptotic DNA fragmentation^24^ and ATM also can be activated by small double-stranded DNA fragments.^50^ Our results suggest that the pan-nuclear *γ*H2AX response is triggered by transient pan-nuclear p-ATM and p-DNA-PKcs signals in resting HPBLs, and that the pan-nuclear *γ*H2AX response has a key role in X-ray-induced apoptosis in resting HPBLs.

*γ*H2AX was recently shown to be a crucial component in modulating apoptosis.^18,51^ Lu *et al.*^51^ showed that H2AX S139 phosphorylation by ultraviolet A-activated c-Jun N-terminal kinase (JNK) is required for DNA ladder formation mediated by caspase-3/caspase-activated DNase (CAD) in apoptotic cells. Cook *et al.*^18^ reported that phosphorylation of tyrosine 142 of H2AX promoted the apoptotic response and inhibited the DDR to DNA damage. When H2AX tyrosine 142 was mutated to alanine, only *γ*H2AX foci, but not pan-nuclear *γ*H2AX and TUNEL staining, occurred after irradiation in H2AX^−/−^ mouse embryonic fibroblast cells transfected with mutant H2AX (Y142F).^18^ These observations support our results that X-irradiation-induced pan-nuclear *γ*H2AX is involved in X-irradiation-induced apoptosis in resting HPBLs. Our results also suggest that there are no H2AX mutations at S139 and Y142 sites in HPBLs, and that pan-nuclear *γ*H2AX staining can be used to detect the apoptosis of HPBLs exposed to low LET IR. In addition, no *γ*H2AX, p-ATM and p-DNA-PKcs foci were observed in the X-ray-induced pan-nuclear response and TUNEL-positive HPBLs. One possible explanation is that the condensed chromatin in the late stages of apoptosis blocks the expression of DNA repair proteins.^19^ All together, we proposed that *γ*H2AX regulates apoptosis in a pan-nuclear response pattern and is involved in the DSB repair response in a focal pattern in resting HPBLs.

It has been reported that IR-induced HPBLs apoptosis is mediated by caspase cascade and is p53-dependent,^42–47^ which raises a question of whether X-irradiation-induced pan-nuclear *γ*H2AX response is also dependent on p53 and caspases. In the present study, we demonstrated that p53 inhibitor PFT-*μ* and caspase inhibitor ZVAD-fmk inhibited the X-irradiation-induced nuclear-wide *γ*H2AX response and apoptosis. A possible explanation is that the pan-nuclear *γ*H2AX response is involved in the initiation and execution of apoptosis in HPBLs, and that p53 and caspases mediate the pan-nuclear *γ*H2AX response by priming and executing apoptosis, respectively. Coincident with the inhibition of X-irradiation-induced pan-nuclear *γ*H2AX response, we found that p53 inhibitor PFT-*μ* decreased the X-irradiation-induced pan-nuclear p-ATM and p-DNA-PKcs responses, suggesting that p53 may mediate the pan-nuclear *γ*H2AX response through activating the pan-nuclear p-ATM and p-DNA-PKcs responses. It has been found that wild-type p53 protein is able to rejoin DNA with DSBs to facilitate precise ligation through direct physical interaction of p53 with DNA-PK of NHEJ,^52–54^ and that p53 phosphorylation by ATM is involved in the initial steps of DNA damage signaling.^55^ In the present study, we also demonstrated that p53 inhibition by PFT-*μ* results in a decrease of DSB repair and an increase of chromosome breaks by inhibiting the X-irradiation-induced p-ATM and p-DNA-PKcs focus formation. Similar to ATM/DNA-PKcs inhibitor KU55933, PFT-*μ* can inhibit the pan-nuclear *γ*H2AX response, the formation of p-ATM and p-DNA-PKcs foci, and pan-nuclear p-ATM and p-DNA-PKcs responses. However, we also found that caspase inhibitor ZVAD-fmk decreased the nuclear-wide *γ*H2AX response and apoptosis but did not affect the X-ray-induced chromosome breaks, suggesting that the nuclear-wide *γ*H2AX response is involved in caspase-mediated apoptotic execution, but caspases are not involved in DSB repair, as caspases are activated at the execution phase of apoptosis when DSB repair process is finished.

It is well established that the IR-induced massive cell death in radiosensitive tissues is not due to irreversible damage of cells but rather to activation of apoptosis, therefore blockage of apoptosis has become a pharmacological approach to radioprotection of normal tissues.^56^ Similarly, inhibition of DSB repair has been well known as an efficient approach to facilitate the apoptosis and sensitize tumor cells to IR and DSB-inducing chemotherapeutic agents such as etoposide, doxorubicin and camptothecin.^57^ Surprisingly, we found that ATM/DNA-PKcs inhibitor KU55933 protected resting HPBLs from the X-ray-induced pan-nuclear *γ*H2AX response and apoptosis, although it inhibited the X-ray-induced DSBs repair and enhanced chromosomal breaks. Our results are consistent with the literature reports that inhibition of ATM and DNA-PKcs phosphorylation by KU55933 can result in the suppression of H2AX phosphorylation and etoposide-induced apoptosis in resting HPBLs.^26,58^ It is believed that HPBLs in a resting status tolerate a limited un-repair and inaccurate repair, and that there are opposite actions of KU55933 on normal resting cells and proliferating cancer cells. Owing to dual functions of p53 in mediating the DSB repair and apoptosis in resting HPBLs, p53 inhibitor PFT-*μ*, like ATM/DNA-PKcs inhibitor KU55933, protected resting HPBLs from the X-ray-induced pan-nuclear *γ*H2AX response and apoptosis, and concurrently induced a decrease of DSB repair and an increase of chromosome breaks and frequencies of chromosomal aberrations. Our observations are consistent with the findings that PFT-*α* decreases the repair efficiency of X-ray-induced DNA lesions, leading to increased frequencies of chromosomal aberrations and reduced apoptosis in HPBLs.^46^ Unlike the dual role of KU55933 and PFT-*μ*, caspase inhibitor ZVAD-fmk protected HPBLs from X-ray-induced nuclear-wide *γ*H2AX response and apoptosis, and did not worsen the X-ray-induced chromosomal instability. More importantly, we found that the inhibition of X-ray-induced nuclear-wide *γ*H2AX response and apoptosis by KU55933, PFT-*μ* and ZVAD-fmk aggravated the inhibitory effects of X-irradiation on the proliferative response of HPBLs to mitogens, suggesting that the inhibition of the nuclear-wide *γ*H2AX response and apoptosis does not protect the function of HPBLs. It is possible that the inhibition of DSB repair by KU55933 and PFT-*μ* does not result in correction of chromosome aberrations in HPBLs, and that the increased genomic instability leads to further suppression of the proliferative response of HPBLs exposed to X-irradiation, which limits the pharmacological use of DSB repair inhibitors and p53 inhibitors in radioprotection of normal tissues. Nevertheless, the aggravated effect of ZVAD-fmk on IR-induced proliferative inhibition may be related to suppression of the expression of lymphocyte-stimulated factor receptors, such as IL-2.^59^ Lawrence *et al.*^59^ found that ZVAD-fmk inhibits the proliferative response of human T lymphocytes induced by mitogens and IL-2 via inhibiting the expression of the IL-2 receptor, and that suppression of human T-cell proliferation by ZVAD-fmk is independent of its caspase inhibition properties, as ZVAD-fmk inhibits caspase processing during apoptosis but not during T-cell activation. Because caspase inhibitors are capable of providing reversible inhibitory effects on proliferative response in HPBLs by temporarily blocking the expression of proliferative response receptors, we proposed that inhibition of caspases would be a promising pharmacological approach for radioprotection of HPBLs.

In summary, we showed herein that X-irradiation induced not only the pan-nuclear *γ*H2AX response but also the transient pan-nuclear p-ATM and p-DNA-PKcs responses in addition to the formation of nuclear DDR foci containing activated ATM, DNA-PKcs and *γ*H2AX in resting HPBLs. The pan-nuclear *γ*H2AX response and the transient pan-nuclear p-ATM and p-DNA-PKcs responses were directly related to X-ray-induced apoptosis. Moreover, the pan-nuclear *γ*H2AX response and apoptosis were mediated not only by transient pan-nuclear p-ATM and p-DNA-PKcs signals but also by p53 and pan-caspase activities in resting HPBLs exposed to X-irradiation. ATM/DNA-PKcs inhibitor and p53 inhibitor attenuate the X-ray-induced pan-nuclear *γ*H2AX response and apoptosis, but worsen the X-ray-induced functional impairment and genomic instability, suggesting that these inhibitors cannot be used as radioprotective agents for HPBLs. The pan-caspase inhibitor attenuates X-ray-induced pan-nuclear *γ*H2AX response and apoptosis with no effect on X-ray-induced genomic instability and holds developmental promise.

## Materials and Methods

### Peripheral blood sample collection and lymphocyte isolation and culture

Human peripheral blood samples were obtained from informed healthy male and female volunteers (22–25 years old), and the research was approved by Fudan University School of Public Health Institutional Review Board. For each experiment, blood was collected in sterile heparinized Vacutainers from three to six different donors. Lymphocytes were isolated from blood using density gradient centrifugation in Ficoll-Paque (Cedarlane Co., Burlington, ON, Canada) according to the manufacturer’s instructions. Isolated HPBLs at a density of 1×10^6^ cell/ml were cultured in RPMI 1640 medium (Gibco, Invitrogen Technologies, Carlsbad, CA, USA) supplemented with 15% fetal calf serum (Invitrogen Technologies), 5% (v/v) plasma, 100 U/ml penicillin and 100 *μ*g/ml streptomycin at 5% CO_2_ and 37°C in a humidified incubator. The viability of the isolated HPBLs was above 95% as measured with the trypan blue exclusion assay.

### Irradiation and treatment

An X-Rad 320 biological irradiator (Precision X-Ray, Inc., North Branford, CT, USA) was used for the X-ray irradiation, and the dose rate was 1.83 Gy/min. The HPBLs were pretreated with 10 *μ*M KU55933 (Selleckchem, Houston, TX, USA) for 2 h, 20 *μ*M PFT-*μ* (Selleckchem) for 5 h or 100 *μ*M ZVAD-fmk (Selleckchem) for 1 h, and then irradiated in a flask at room temperature at doses of 0.5, 2 or 5 Gy. After irradiation, the cells were incubated in a CO_2_ incubator at 37 °C and harvested after various repair periods for immunofluorescence staining, apoptosis assay and western blotting analysis. For the lymphocyte proliferative assay, phytohemagglutinin (Shanghai Jikong Biotechnology, Shanghai, China) was added to the culture medium after IR, and the cells were further incubated for 48 h.

### Immunofluorescence analysis

The suspension of irradiated and sham-irradiated HPBLs was immediately placed on ice for 10 min and washed in ice-cold PBS, and the cells were spun onto the triple chambered slides precoated with 50 mg/l poly-L-lysine (Sigma, St. Louis, MO, USA) using a cytospin centrifuge (Iris sample processing, Beckman Coulter Inc., Miami, FL, USA), fixed with 4% paraformaldehyde for 15 min, permeabilized with 0.5% Triton X-100 for 15 min and followed by blocking with 10% (v/v) fetal calf serum in PBS at 37 °C for 1 h. The cells were then incubated overnight at 4 °C with anti-*γ*H2AX (S139) antibody (Cell Signaling Technology, Danvers, MA, USA), anti-phospho-ATM (S1981) antibody (Abcam, Hong Kong, China) and anti-phospho-DNA-PKcs (T2609) antibody (Abcam) or anti-Ki67 antibody (Cell Signaling Technology), followed by secondary antibody labeled with Alexa Fluor 488-conjugated donkey anti-rabbit (Molecular probe, Life Technologies, Grand Island, NY, USA), Alexa Fluor 555-conjugated donkey anti-mouse or Alexa Fluor 647-conjugated donkey anti-rabbit (Molecular probe) at room temperature for 1 h. Fluorescence images were acquired by an Olympus BX51 fluorescent microscope (Olympus Optical Co., Tokyo, Japan) equipped with a COHU CCD camera (Audio Video Supply, San Diego, CA, USA) and VideoTesT-FISH software (VideoTesT, Saint Petersburg, Russia). Images used for comparisons between different treatments were acquired and processed with the same instrument settings. The normalized levels of pan-nuclear staining or foci of *γ*-H2AX, p-ATM (S1981) and p-DNA-PKcs (T2609) were the ratios of the number of pan-nuclear staining or foci to the number of cells scored. In addition, colocalization of TUNEL staining with pan-nuclear *γ*-H2AX, p-ATM or p-DNA-PKcs staining, and colocalization of pan-nuclear *γ*-H2AX staining with pan-nuclear p-ATM or p-DNA-PKcs staining were examined. The colocalization ratios were calculated as follows: number of colocalized pan-nuclear staining/number of TUNEL staining or pan-nuclear *γ*-H2AX staining.

### Apoptosis detection

After pretreatment with inhibitors of ATM/DNA-PKcs, p53 and pan-caspases or/and X-irradiation, the cell monolayers were spun down on slides, and subjected to terminal deoxynucleotidyl transferase-mediated dUTP (2′-deoxyuridine 5′-triphosphate) biotin nick end labeling (TUNEL) staining using a TUNEL Apoptosis Detection Kit (Vazyme Biotech, Nanjing, Jiangsu Province, China) for *in situ* detection of apoptosis according to the manufacturer’s instructions. For combination with immunofluorescence analysis, fluorescein isothiocyanate-12-dUTP labeling mix was added and incubated for 1 h after incubation with the primary antibody, followed by incubation with the secondary antibody. The TUNEL-stained cells were counter-stained with mounting medium containing 4′,6-diamidino-2-phenylindole (DAPI) (Santa Cruz Biotechnology, Santa Cruz, CA, USA); the fluorescein isothiocyanate-labeled TUNEL-positive cells were imaged using an Olympus BX51 fluorescent microscope, and the TUNEL-positive cells were counted to calculate the TUNEL indices.

### Chromosomal aberration analysis

After pretreatment with inhibitors of ATM/DNA-PKcs, p53 and pan-caspases, HPBLs were irradiated by X-rays. After a 2-h repair period, the cells were incubated with colcemid and phytohemagglutinin, and the levels of X-ray-induced chromosomal aberrations in HPBLs were determined at 48 h after irradiation. Chromosome preparations were made using standard techniques after the incubation of the cells in hypotonic solution (0.075 M KCl) and fixation with methanol/acetic acid (3 : 1). For each sample, 100 metaphases per donor were scored. The chromosomal aberrations were classified according to Savage.^60^

### Western blotting

Cell extracts were prepared in radioimmunoprecipitation assay buffer containing 1% (v/v) proteinase inhibitor, 1% (v/v) phosphatase inhibitor cocktail, 1 mM phenylmethylsulfonyl fluoride and 1 mM sodium orthovanadate. Aliquots (60 *μ*g protein) from each sample were loaded into each lane of sodium dodecyl sulfate-polyacrylamide gel electrophoresis gels and subjected to gel electrophoresis, and followed by transfer to polyvinylidene fluoride membranes (Millipore, Bedford, MA, USA). The membranes were incubated overnight at 4 °C with the primary anti-*γ*-H2AX (S139) antibody (1 : 1000) (Cell Signaling Technology), anti-phospho-ATM (S1981) antibody (1 : 1000) (Abcam), anti-ATM antibody (1 : 500) (Santa Cruz), anti-phospho-DNA-PKcs (T2609) antibody (1 : 1000) (Abcam), anti-DNA-PCcs antibody (1 : 500) (Santa Cruz), anti-phospho-p53 (S15) antibody (1 : 1000) (Cell Signaling Technology), anti-p53 antibody (1 : 500) (Santa Cruz) or anti-cleaved-caspase-3 antibody (1 : 1000) (Cell Signaling Technology). The membranes were then incubated for 1 h at room temperature with the secondary antibody: IgG-HPR antibody (1 : 2500) (Immunology Consultants Laboratory, Inc., Portland, OR, USA). Enhanced chemiluminescence was then performed (ECL Kit, Millipore) according to the manufacturer's protocol. The densitometry analysis was performed with the ChemiDoc detection system (Bio-Rad Laboratories, Hercules, CA, USA) and Quantity One software (Bio-Rad). The membranes were also probed with anti-GAPDH antibody (1 : 1000) (Huaan Biotechnology, Hangzhou, Zhejiang Province, China), anti-*β*-actin antibody (1 : 5000) (Huaan Biotechnology) or anti-vinculin antibody (1 : 10000) (Abcam) as the internal control.

### Statistical analysis

Data are presented as the means±standard deviation. Statistical analysis of the data was performed using SPSS 17.0 software. Independent-samples *T* test was applied to analyze the differences. A difference with *P*<0.05 was considered to be statistically significant.

## Figures and Tables

**Table 1:** Effects of KU55933, PFT-*μ* and ZVAD-fmk on frequencies of X-irradiation-induced chromosomal aberrations at 48 h post irradiation in resting HPBLs

*Treatment*	*Chromosomal aberrations/100 cells*		*% Aberrant cells*
	*Breaks*	*Dicentrics+rings*	
Ctrl	0.7±0.3	0.5±0.5	1.2±0.8
KU55933	0.7±0.6	0	0.7±0.6
PFT-*μ*	0	0	0
ZVAD-fmk	0.3±0.6	0	0.3±0.6
IR	13.8±2.5**	14.1 ±2.2**	21.3±3.3**
KU55933+IR	24.2±2.3**^§§^	23.7±3.7***^§^	40.7±5.0**^§§^
PFT-*μ*+IR	22.3±2.4***^§^	20.7±2.8**^§^	34.7±2.4**^§§^
ZVAD-fmk+IR	17.9±5.8*	13.5±4.9*	25.6±4.8**

Resting HPBLs were pretreated with 10 *μ*M KU55933 for 2 h, 20 *μ*M PFT-*μ* for 5 h or 100 *μ*M ZVAD-fmk for 1 h followed by irradiation with 2 Gy X-rays, and incubated with PHA and colcemid for 48 h. The metaphase cells were then harvested, and a total of 100 metaphase cells were analyzed. The values represent the means±S.D. of HPBLs from three individual donors. *P<0.05, **P<0.01 and ***P<0.001, compared with non-irradiated control and inhibitor treatment alone; ^§^ *P*<0.05 and ^§§^ *P*<0.01, compared with IR alone.

## Abbreviations

HPBLs: human peripheral blood lymphocytes
IR: ionizing radiation
*γ*H2AX: Phosphorylated histone variant H2AX
dUTP: 2′-deoxyuridine 5′-triphosphate
TUNEL: terminal deoxynucleotidyl transferase-mediated dUTP biotin nick end labeling
LET: linear energy transfer
p-ATM: phosphorylated Ataxia telangiectasia mutated
p-DNA-PKcs: phosphorylated DNA-dependent protein kinase catalytic subunit
ATM: Ataxia telangiectasia mutated
DNA-PKcs: DNA-dependent protein kinase catalytic subunit
PFT-*μ*: Pifithrin-*μ*
PFT-*α*: Pifithrin-*α*
DSBs: double strand breaks
DDR: DNA damage response
TRAIL: tumor necrosis factor-related apoptosis-inducing ligand
JNK: c-Jun N-terminal kinase
CAD: caspase-3/caspase-activated DNase
NHEJ: non-homologous end-joining
IL-2: Interleukin-2
RPMI: Roswell Park Memorial Institute
PHA: phytohemagglutinin
PBS: phosphate-buffered saline
DAPI: 4′,6-diamidino-2-phenylindole
S.D.: standard deviation.
